# Herbicide Resistance in Plants

**DOI:** 10.3390/plants9040435

**Published:** 2020-04-01

**Authors:** Hugh J Beckie

**Affiliations:** Australian Herbicide Resistance Initiative (AHRI), School of Agriculture and Environment, The University of Western Australia, Perth, WA 6009, Australia; hugh.beckie@uwa.edu.au

**Keywords:** herbicide resistance, non-target-site resistance, precision weed management, resistance management, weed biology, weed genomics

## Abstract

Herbicide resistance in weeds is perhaps the most prominent research area within the discipline of weed science today. Incidence, management challenges, and the cost of multiple-resistant weed populations are continually increasing worldwide. Crop cultivars with multiple herbicide-resistance traits are being rapidly adopted by growers and land managers to keep ahead of the weed resistance tsunami. This Special Issue of *Plants* comprises papers that describe the current status and future outlook of herbicide resistance research and development in weedy and domestic plants, with topics covering the full spectrum from resistance mechanisms to resistance management. The unifying framework for this Special issue, is the challenge initially posed to all of the contributors: what are the (potential) implications for herbicide resistance management?

## 1. Introduction

Since the first global cases of herbicide resistance in weeds in the late 1950s, there are now over 500 unique cases reported in non-cropland and almost 100 different crops in 70 countries—over 260 species compromising the efficacy of over 160 herbicides or over 20 herbicide sites of action (SOA) [1]. The current rate of increase in the number of weed species resistant to glyphosate (e.g., see Alcántara-de la Cruz et al. [2] this issue) is second only to that of acetolactate synthase inhibitors. Since first introduced in the early 1980s, cultivars of major agronomic field crops possessing herbicide-resistance traits now occupy a significant proportion of the global crop production area [3,4]. This Special Issue presents a collection of papers that highlight the continuing breadth and depth of basic and applied herbicide resistance research and development in both weedy and crop species. As the privileged guest editor, I share my perspectives on key messages, and future directions gleaned from these volunteered or invited contributions.

## 2. Key Messages

An integral element of herbicide resistance surveillance is the periodic sensitivity analysis of populations of a weed species in an agroregion to commonly used herbicides. Such an analysis provides information on the inter- and intra-population variability in the effective dose (ED) required for 50 or 90%, etc., reduction in survival or biomass. Therefore, sensitivity analysis can determine if populations are becoming less sensitive to a herbicide over time, and if label rates need to be adjusted accordingly. These foundational studies are extremely important in mitigating quantitative (creeping) resistance evolution, particularly for key herbicides such as glyphosate and major problematic outcrossing weeds such as *Lolium* spp. [5,6]. Intentional or unintentional sublethal herbicide doses may even alter the metabolism, growth, and survival of susceptible plants of highly-selfing species, such as demonstrated for *Avena fatua* L. (wild oat) [7].

Target-site mutations conferring evolved herbicide resistance in weeds are known in nine different herbicide SOA. An emerging trend is increased cases of multiple mutations, including multiple amino acid changes at the glyphosate target site as well as mutations involving two nucleotide changes at a single amino acid codon [8]. Non-target-site resistance (NTSR) to herbicides in weeds, such as enhanced metabolism by P450 monooxygenases, is an increasingly serious threat to sustainable weed management as the efficacy of multiple SOA herbicides may be compromised. Although much more difficult to investigate than target-site resistance, steady advances are being made in the physiological, biochemical and molecular basis of NTSR mechanisms in weeds [9].

The fields of genomics, transcriptomics, proteomics, and metabolomics—collectively referred to as ‘omics’—describe the component parts of the biological system that lead to the presentation of traits. Unravelling the genome of major global weedy species will greatly facilitate the identity and function of major and minor genes responsible for herbicide resistance [10]. Draft weed genomes can provide insights on the evolutionary origins of weeds, allowing identification of management practices that may mitigate resistance evolution. Moreover, genomics can identify strengths and weaknesses of weed populations that can be targeted for control, while providing fundamental information on how plants rapidly respond to herbicide selection. The weed omics era of today is enabling translational research to bridge from basic science to field applications, by linking systems-scale science to applied science for practitioners [11]. Weed science is still learning how to integrate omics technologies into the discipline; however, omics techniques are more frequently being implemented in novel ways to address basic questions in weed biology or practical questions of improving weed management; for the latter, the potential benefits of weed omics will be best realized for farms utilizing advanced data science approaches necessary for the implementation of digital farming [11]. 

After a 35-year hiatus in the commercialization of new SOA herbicides, there is now optimism in the agri-chemical industry as new SOA herbicides are being introduced for control of key economic weeds in major agronomic crops. A review in this issue of the current status and future prospects in herbicide discovery offer insights into novel potential target sites in plants and innovative approaches or processes to facilitate new herbicide SOA discovery [12]. Because of this hiatus in SOA discovery and commercialization, cultivars of the major agronomic crops, particularly maize (*Zea mays* L.) and soybean (*Glycine max* L. Merr.), are being conventionally bred or genetically engineered with combined (stacked) pesticide-resistance traits. A review in this issue summarizes their current status and future outlook [13]. Recent global developments and trends in herbicide resistance management also include the increasing reliance on pre-emergence vs. post-emergence herbicides because of weed resistance, breeding for weed-competitive cereal crop cultivars, expansion of harvest weed seed control practices, and advances in site-specific or precision weed management (via prescription maps or in real-time) [14]. 

## 3. Future Directions

Natural selection for herbicide-resistant weed genotypes may act on standing genetic variation or on a genetic and physiological background that is altered because of stress responses to sublethal herbicide exposure. Stress-induced changes include DNA mutations, epigenetic alterations, transcriptional remodeling, and protein modifications, all of which can lead to herbicide resistance and various pleiotropic effects [15]. Studies examining stress-induced evolution of herbicide resistance and related pleiotropic effects are needed to inform improved herbicide-resistant weed prevention and management strategies [7]. As both the incidence of weed populations with NTSR and the worldwide occurrence of environmental stress are expected to increase, expanded research on NTSR evolution and its potential for pleiotropic effects should be a high priority [15].

A primary goal driving the need to characterize herbicide resistance mechanisms is the management of herbicide-resistant weeds. Better understanding is needed of the relationship between target-site resistance mutations or mechanisms in troublesome weed species, their geographic distribution and prevalence across an agroregion, resulting in cross-resistance patterns, and associated fitness costs. Continuing advances or improvements are expected in the efficiency and accuracy of high throughput in vitro diagnostic techniques [16]. A uniform and replicable system for in planta functional validation, which is the gold standard for demonstrating resistance and susceptibility, is necessary to facilitate high-throughput screening initiatives [8]. Because the evolution of NTSR via herbicide metabolism is a serious threat to weed management, identification of the genes endowing resistance and their functional characterization are important future research goals for possible mitigation and management strategies. The increasing availability of sequenced genomes for different weed species will greatly accelerate research in this area [10]. Accurately assessing fitness costs of resistance and deriving practical management tactics to potentially exploit this phenomenon in resistant weed populations will continue to be an important research endeavour [17]. 

Omics research in weed science faces several challenges, including management of large and complex omics datasets, efficient and accurate annotation of reference genome assemblies and eventual pan genomes, and the large number of weed species with a diversity of weedy traits and variation in evolutionary strategies. Examining the diverse ways that researchers working in model systems use omics technologies in their respective fields can provide established tools and templates to address the future needs of the weed science community [11]. In particular, method standardization for utilizing next generation sequencing in weed science, improving herbicide resistance diagnostics with omics, and improved gene function validation for herbicide resistance mechanisms are attainable medium-term (5 to 10 year) goals. Can we alter weed populations to make them easier to control? Current and future omics tools to improve herbicide-resistant weed management, such as gene drive systems for sensitizing herbicide-resistant weed populations, requires proof of concept studies but has promising long-term potential [10,11,18]. A better understanding of weed species at the population, genomic, and genic levels using population genomic approaches will help begin to address that question.

Ultimately, basic or applied herbicide resistance research and development should inform resistance management by growers and land managers. Sustaining the utility of existing herbicides and effective stewardship guidelines for herbicide-resistant crops will continue to demand innovative research and development to address these challenges. Adoption of some recommended best management practices by end-users may require private or public sector financial incentives. Recent advances in precision or digital agriculture have largely been driven by significant private-sector investments. It offers the best route for optimizing crop production and crop protection across a field by varying input levels commensurate with site-specific soil or environmental conditions that govern yield potential. The ongoing challenge is the development of user-friendly and cost-effective technologies or systems that can be easily integrated into existing farming enterprises.

## References

[1] Heap I.M. (2020). International Survey of Herbicide Resistant Weeds. http://www.weedscience.org.

[2] Alcántara-de la Cruz R., Domínguez-Martínez P.A., da Silveira H.M., Cruz-Hipólito H.E., Palma-Bautista C., Vázquez-García J.G., Domínguez-Valenzuela J.A., de Prado R. (2019). Management of glyphosate-resistant weeds in Mexican citrus groves: Chemical alternatives and economic viability. Plants.

[3] Beckie H.J., Harker K.N., Hall L.M., Warwick S.I., Légère A., Sikkema P.H., Clayton G.W., Thomas A.G., Leeson J.Y., Séguin-Swartz G. (2006). A decade of herbicide-resistant crops in Canada. Can. J. Plant Sci..

[4] Green J.M., Owen M.D.K. (2011). Herbicide-resistant crops: Utilities and limitations for herbicide-resistant weed management. J. Agric. Food Chem..

[5] Panozzo S., Collavo A., Sattin M. (2020). Sensitivity analysis of Italian *Lolium* spp. to glyphosate in agricultural environments. Plants.

[6] Busi R., Powles S.B. (2009). Evolution of glyphosate resistance in a *Lolium rigidum* population by glyphosate selection at sublethal doses. Heredity.

[7] Tafoya-Razo J.A., Oregel-Zamudio E., Velázquez-Márquez S., Torres-García J.R. (2019). 10,000-times diluted doses of ACCase-inhibiting herbicides can permanently change the metabolomic fingerprint of susceptible *Avena fatua* L. plants. Plants.

[8] Murphy B.P., Tranel P.J. (2019). Target-site mutations conferring herbicide resistance. Plants.

[9] Jugulam M., Shyam C. (2019). Non-target-site resistance to herbicides: Recent developments. Plants.

[10] Martin S.L., Parent J.-S., Laforest M., Page E., Kreiner J.M., James T. (2019). Population genomic approaches for weed science. Plants.

[11] Patterson E.L., Saski C., Küpper A., Beffa R., Gaines T.A. (2019). Omics potential in herbicide-resistant weed management. Plants.

[12] Dayan F.E. (2019). Current status and future prospects in herbicide discovery. Plants.

[13] Nandula V.K. (2019). Herbicide resistance traits in maize and soybean: Current status and future outlook. Plants.

[14] Beckie H.J., Ashworth M.B., Flower K.C. (2019). Herbicide resistance management: Recent developments and trends. Plants.

[15] Dyer W.E. (2018). Stress-induced evolution of herbicide resistance and related pleiotropic effects. Pest Manag. Sci..

[16] Délye C., Michel S., Pernin F., Gautier V., Gislard M., Poncet C., Le Corre V. (2020). Harnessing the power of next generation sequencing technologies to the purpose of high-throughput pesticide resistance diagnosis. Pest Manag. Sci..

[17] Vila-Aiub M.M. (2019). Fitness of herbicide-resistant weeds: Current knowledge and implications for management. Plants.

[18] Neve P. (2018). Gene drive systems: Do they have a place in agricultural weed management?. Pest Manag. Sci..

